# Ovarian Tumor

**Published:** 1865-09

**Authors:** 


### Ovarian Tumor.

George T. Elliot, M. D., Professor in Bellevue Hospital Medical College communicates to the New York Medical Journal, the report of an operation for removal of an oxogenous ovarian tumor, weighing seven pounds, with fatal results. The operation, circumstances, attendants, time, symptoms, treatment, and everything connected with it, is reported with the most painful exactness; there is but one single omission to be conceived; he says, "the first incision was made at 2.16 P. M. with a broad scalpel." The size of the scalpel with which he operated is given, but the name of the manufacturer is omitted. Dr. Elliot makes some very pertinent remarks upon the importance of the operation, the value of reporting fatal cases and the great impropriety of operating under unfavorable circumstances. The report is instructive, but throughout the whole report we have never met with a minuteness of detail so remarkable.

